# Acute Poliomyelitis in New York

**Published:** 1909-07-31

**Authors:** 


### SPECIAL ARTICLES.

### ACUTE POLIOMYELITIS IN NEW YORK.

OF all the infantile disorders whose diagnosis and origin baffle not infrequently even the most expert, acute anterior poliomyelitis is one of the most mysterious.

One of the most characteristic and unaccounted-for features of this condition is its occasional outbreak into epidemic form; though, granted an infective causal organism, perhaps it should really excite more surprise that the disease is usually so very sporadic. At all events, the epidemic which last year visited the United States, particularly New York, throws into the shade the records of previous similar epidemics in Stockholm and elsewhere, for over 1,200 cases were reported to the New York City and State Boards of Health, and Dr. Holt says the total is not far from 3,000. The lessons of this extensive visitation are considered in the *Archives of Pædiatrics* by Dr. Henry Koplik and Dr. L. E. La Fetra respectively, from whose articles most of the facts herein set down are derived.

The cases were distributed throughout the city, regardless of the sanitary state of the various districts or the social position of the inhabitants. Water and milk supplies and hygiene in general are believed to have been better than in any previous year, and fashionable suburbs suffered equally with crowded slums and better-class urban districts. The epidemic affected children of all ages from infancy to adolescence, and a few scattered cases occurred among adults. The disease is described as conforming to three fairly distinct types, two of which differed very materially from the usual text-book account of acute anterior poliomyelitis. At the onset the symptoms often resembled somewhat those of meningitis, yet not sufficiently so to deceive acute and painstaking diagnosticians. Vomiting was quite common, and occasionally persisted for days, but as a rule occurred only at the onset.

Convulsions were the exception, though fever was practically the rule; the temperature ranged from 100° to 102° F. in mild cases, and up to 105° in severe ones. Cough, tonsillitis, and sore throat were rather rare; rigidity of the neck a good deal less so. Irritability and restlessness were very common, and general hyperæsthesia was occasionally recorded. Headache, sometimes of an excruciating kind, was often noted, and pain or tenderness in the affected limbs was very usual indeed. Both writers emphasise this last point, as the distribution along the course of peripheral nerves caused much confusion with neuritis; the resemblance to early meningitis is also sufficiently obvious from this brief list of premonitory symptoms.

Dr. Koplik's three types are described as cerebral, neuritic, and the ordinary sporadic form in which paralysis is almost the first sign of the disorder. In the cerebral cases a child in apparent health would either go suddenly into a condition of unconsciousness, or would awake complaining of headache and vomiting, and then either become comatose or develop paralysis of all four limbs without losing consciousness; the temperature also rose, more or less. In bad cases symptoms referable to the bulb supervened, and paralysis of the respiratory muscles and of those of deglutination would soon be followed by death from bulbar paralysis. In other cases these cerebral symptoms would abate, though the paralysis of the extremities persisted, accompanied sometimes by paralysis of the thoracic and abdominal muscles. Rigidity of the neck and even head retraction were occasionally noticed.

In the neuritic type acute pains in the extremities, often referred to the joints, first attracted attention. Gradually paralysis of one or more limbs developed, with the onset as a rule of febrile disturbance also; but this initial pyrexia was soon succeeded by normal readings. Rheumatism was simulated in many cases, and neuritis in others. The pains sometimes lasted as long as three or four weeks, and complete, or almost complete, recovery from the paralysis seems to have been comparatively common in these neuritic cases. The third and more classical type scarcely needs detailed description: a slight stomachic or intestinal disturbance, and febricula, followed by paralysis of one or two extremities, or of a group of muscles of one limb only, was the usual clinical picture.

With regard to the causation of this acute and curious disease, many observers resorted to lumbar puncture; but one and all failed to discover either in films or cultures any organism, though a polynuclear leucocytosis was occasionally found. The cerebro-spinal fluid was uniformly clear, and as a general rule was not exuded under any abnormal pressure. A moderate leucocytosis in the blood is described by La Fetra. The conceptions of the pathology which have been originated in Scandinavia by Harbitz, Scheele, Medin, and Wickham during recent years seem to commend themselves strongly to American physicians. The process is regarded as an acute disease of the whole central nervous system, characterised especially by minute hæmorrhages. These may occur not only in the anterior columns and horns of the cord, but in the posterior; not only in the spinal cord, but in the pons, medulla, or cortex. According to their principal distribution will result a cortical or pontine, encephalitis, a spinal poliomyelitis, and so on. The neuritic cases are explained as due to irritation of the peripheral nerves resulting from inflammation of the *pia mater* of the cord and brain, which is believed always to precede that of the grey matter of the cord or cortex. Dr. Flexner concludes from the negative bacteriological findings of the cerebro-spinal fluid examinations that a toxic degeneration due to toxins produced elsewhere in the body is a more plausible suggestion than an acute microbic infection of the cord itself.

It is particularly evident that the New York physicians, equally with those in Norway and Sweden who have had the opportunity of studying epidemics, are no longer inclined to regard the disease as one essentially affecting the anterior horn cells. The deep reflexes were very often noted to be not entirely lost, as they necessarily are in a destructive lesion of those cells, such as occurs in the ordinary form of acute anterior poliomyelitis. It follows logically enough that this last name is somewhat a misnomer, inasmuch as many other parts of the central nervous system may be affected by the disease process. In the neuritic cases the knee-reflexes were nearly always increased, and even in some of the cases resembling the classical text-book descriptions they were present, or not entirely lost. In the cerebral cases the reflexes were as a rule obtained, but there was no Kernig sign nor a Babinsky reflex elicited: so reports Koplik, but La Fetra found the latter sign three times in twenty babies, and never saw an exaggerated tendon reflex throughout the epidemic. Dr. Jacobi remarks that where polioencephalitis predominates the patellar reflex should remain intact: when poliomyelitis is the main lesion, the reflex rapidly disappears and atrophy will soon set in. He regards the prognosis of the former as much better than that of the latter. Spastic paralyses were seen in a few cases, though the great majority were of the usual flaccid character. It was also especially noticed that in those patients in whom the paralysis was to remain permanent the atrophy of muscle, and the onset of degeneration and of contractures were extraordinarily rapid. Instances of paralysis affecting the face alone were by no means rare, and several of them were at first diagnosed as tuberculous meningitis.

